# Linking social motivation, general motivation, and social cognition to interpersonal functioning in schizophrenia: insights from exploratory graph analysis

**DOI:** 10.1007/s00406-023-01733-4

**Published:** 2023-12-19

**Authors:** Michal Hajdúk, Samuel J. Abplanalp, Amy M. Jimenez, Melissa Fisher, Kristen M. Haut, Christine I. Hooker, Hyunkyu Lee, Joseph Ventura, Mor Nahum, Michael F. Green

**Affiliations:** 1 Department of Psychology, Faculty of Arts, Comenius University in Bratislava, Gondova 2, Bratislava, 811 02 Slovakia; 2 The Centre for Psychiatric Disorders Research, Science Park, Comenius University in Bratislava, Bratislava, Slovakia; 3 Department of Psychiatry, Faculty of Medicine, Comenius University in Bratislava, Bratislava, Slovakia; 4 Department of Veterans Affairs, Desert Pacific Mental Illness Research, Education, and Clinical Center, Los Angeles, USA; 5 Department of Psychiatry and Biobehavioral Sciences, Jane and Terry Semel Institute for Neuroscience and Human Behavior, UCLA, Los Angeles, USA; 6 Department of Psychiatry and Behavioral Sciences, University of Minnesota, Minneapolis, MN USA; 7 Department of Psychiatry and Behavioral Sciences, Rush University Medical Center, Chicago, IL USA; 8 Department of Research and Development, Posit Science Inc, San Francisco, USA; 9 School of Occupational Therapy, Faculty of Medicine, Hebrew University, Jerusalem, Israel

**Keywords:** Motivation, Approach, Avoidance, Interpersonal functioning, Social cognition, Network analysis, Schizophrenia

## Abstract

Motivation in general, and social motivation in particular are important for interpersonal functioning in individuals with schizophrenia. Still, their roles after accounting for social cognition, are not well understood. The sample consisted of 147 patients with schizophrenia. General motivation was measured using the Behavioral inhibition/activation scale (BIS/BAS). Social motivation was measured by Passive social withdrawal and Active social avoidance items from PANSS. Interpersonal functioning was evaluated with Birchwood’s Social Functioning Scale (SFS). We used Exploratory Graph Analysis for network estimation and community detection. Active social avoidance, passive social withdrawal, and social withdrawal/engagement (from SFS) were the most important nodes. In addition, three distinct communities were identified: Social cognition, Social motivation, and Interpersonal functioning. Notably, the BIS and BAS measures of general motivation were not part of any community. BAS showed stronger links to functioning than BIS. Passive social withdrawal was more strongly linked to interpersonal functioning than social cognitive abilities. Results suggest that social motivation, especially social approach, is more closely related to interpersonal functioning in schizophrenia than general motivation. In contrast, we found that general motivation was largely unrelated to social motivation. This pattern highlights the importance of type of motivation for understanding variability in interpersonal difficulties in schizophrenia.

## Introduction

Difficulties in establishing and maintaining close interpersonal relationships are highly prevalent in schizophrenia [1–3]. However, these deficits are not fully explained by core illness features, including symptom severity [4] or nonsocial cognitive deficits [5]. Instead, an emerging line of work indicates that social motivation (i.e., the drive to form and maintain social bonds) [2, 6] and impairments in social cognition [7, 8] are key determinants of successful interpersonal interactions.

Motivation can be divided into two large categories, general and social. In turn, social motivation can be further parsed into two domains: social approach and social avoidance [9]. These domains have different relationships with different types of symptoms. For example, first-episode schizophrenia patients with high levels of social avoidance are more socially anxious [10] and tend to have elevated levels of persecutory delusions [11]. As such, social avoidance could cause one to avoid social situations that are perceived as threatening, ultimately leading to social disconnection. In contrast, reduced social approach resulting in social withdrawal reflects the opposite side of the social motivation continuum. Reduced social approach is considered a core negative symptom [12] as it clusters with avolition and apathy [6]. In addition, people with low social approach/high withdrawal show reduced interest in being with others but may not find social situations inherently threatening. The high rates of social disconnection in schizophrenia may stem from the combination of these two broad social motivation deficits. Unfortunately, interpersonal functioning measures usually do not distinguish the *type* of social motivation behind observed social impairments. Additionally, it is critical to parse the unique effects of social motivation on interpersonal functioning from the effects of general motivation.

General motivation is often assessed according to behavioral inhibition and behavioral activation systems (BIS/BAS [13]). BIS and BAS are distinct neurobiological systems that organize and activate different dispositional or trait-based motivational behaviors. BIS is linked to general avoidance motivation, and BAS to general approach motivation [14], a distinction that is analogous to the one within social motivation. Previous studies (e.g. [15, 16] have found that individuals with schizophrenia show higher BIS scores compared to control participants, indicating elevated threat sensitivity. Therefore, we might expect an individual’s self-reported BIS ratings to be associated with active social avoidance and low levels of self-reported BAS associated with elevated social withdrawal. One prior study [17] used cluster analysis to demonstrate that different combinations of BIS/BAS sensitivities are associated with distinct motivational deficits leading to social disconnection.

Besides motivational impairment, individuals with schizophrenia show robust difficulties in processing of social stimuli. Impaired social cognitive abilities are present from the early course of schizophrenia [18, 19] and manifest as difficulties in social cue perception, such as reading emotions from facial expression and vocal prosody, making inferences about other people’s intentions and behavior, social cognitive biases, and emotion regulation difficulties [20, 21]. Social cognition, therefore, spans both low level perceptual and higher order inferential abilities. Given the complex nature of social interactions and building close interpersonal bonds, impairments in these social cognitive processes may lead to difficulties integrating socially. Indeed, evidence suggests that social cognitive impairments are robust predictors of interpersonal and community functioning [22]. However, whether these associations remain after accounting for impairments in motivation remains unclear.

Social cognition, social motivation, and general motivation may interact in a complex system that contributes to interpersonal functioning. It would be valuable to examine these variables' unique effects on interpersonal functioning while parsing out the effects of other variables. One method that offers a way to deal with complex interdependencies is a network approach [23]. Recently, several studies [24–26] analyzed data from large samples using a network approach and found that variables spanning social cognition, neurocognition and clinical symptoms predict different aspects of real world functioning in schizophrenia. Unfortunately, these studies did not specifically look at the role of state and trait approach/avoidance motivation. Exploratory Graph Analysis (EGA) [27] is a psychometric network model that does not rely on a priori assumptions (e.g., is data-driven). The model uses partial correlations represented as edges and allows for estimating simultaneous associations within and between constructs. EGA can estimate *communities *(i.e., variables that are highly connected and poorly connected to other variables), which provide information on the associations between variables by showing how they cluster.

The current study used a psychometric network approach to examine links between social cognition, social motivation, general motivation, and interpersonal functioning in people with schizophrenia. The research questions for the current set of data-driven analyses were: 1) whether social cognition (emotional recognition, empathic accuracy, emotional intelligence) and social motivation (i.e., social approach and social avoidance), would show unique associations with interpersonal functioning controlling for other variables, 2) whether general motivation, as conceptualized by BIS and BAS, would show any connections with interpersonal functioning, and 3) whether social and general motivation would be highly linked and form one community.

## Methods

### Participants

This study is a secondary analysis of baseline data from a double-blind, multi-site randomized controlled trial that compared the efficacy of a computerized social cognitive training to a control treatment [28]. Participants included clinically stable patients diagnosed with schizophrenia and were recruited from one of the following sites: San Francisco VA Medical Center (SFVAMC), University of Minnesota (UMN), University of California, Los Angeles (UCLA), VA Greater Los Angeles (GLA) and Rush University. Schizophrenia diagnoses were confirmed using the Structured Clinical Interview for DSM-IV (SCID-P) [29]. Additional inclusion criteria included: being between 18 and 65 years of age; estimated IQ ≥ 70 based on the Wechsler Test of Adult Reading (WTAR) [30]; clinically stable for 8 weeks prior to consent; a moderate severity rating or less on hallucinations and unusual thought content (i.e., a score of ≤ 4 on the Positive and Negative Syndrome Scale (PANSS) [31]; and no active suicidal ideation as measured by the Columbia-Suicide Severity Rating Scale (C-SSRS) [32];. Institutional review board approval was obtained at the coordinating center (WIRB Pro Number 20141695; ClinicalTrials.gov Identifier: NCT02246426) and at each site. All participants signed an informed consent prior to study participation.

### Measures

#### General motivation

We used the Behavioral inhibition/activation scales (BIS/BAS) [33] to measure general, trait-based motivation. BIS/BAS is a 20-item self-report scale with seven items corresponding to BIS (e.g., “I feel worried when I think I have done poorly at something”), and 13 total BAS items split into three subscales (Reward Responsiveness: e.g., “When I’m doing well at something I love to keep at it”; Drive: e.g., “When I want something I usually go all-out to get it”; Fun Seeking: e.g., “ I often act on the spur of the moment”). Each BIS/BAS item is rated on a four-point Likert scale, with 1 indicating *very true for me* and 4 indicating *very false for me*. To aid interpretation, items were reverse coded, such that 1 indicates *very false for me* and 4 indicates *very true for me.* For this study, we used two total scores, with one reflecting the sum of BIS items and the other reflecting the sum of BAS items.

#### Social motivation

Measures of state-based social motivation were drawn from the PANSS [31]—a clinical, interview-based measure that includes items reflecting three symptom dimensions: positive, negative, and general psychopathology. All items are rated on a 1 (*absent*) to 7 (*extreme*) scale, with higher scores indicating greater severity. For this study, we used an item from the negative dimension that assesses *passive/apathetic* social withdrawal (i.e., diminished interest and initiative in social interactions due to passivity, apathy, energy or avolition) and an item from the general psychopathology dimension that assesses *active* social avoidance (i.e., diminished social involvement associated with unwarranted fear, hostility, or distrust). We chose those items as our measures of social approach and avoidance motivation.

#### Social cognition

We utilized three well-established social cognitive tasks that were among those included in the social cognitive composite used in the RCT study. We selected tasks that tap into both lower (i.e., perceptual) and higher (i.e., inferential) order processes.

1. Penn Emotional Recognition Test (ER-40) [34]. This task assesses categorical identification of facial emotions. It requires participants to appropriately label photos of facial emotions from five possible choices (anger, fear, happiness, sadness, or no emotion). Forty trials are completed, including four low-intensity and four high-intensity photos for each emotion, and eight neutral expressions photos. Total number of correct responses were used in the further analysis.
2. Empathic Accuracy Task (EA Task) [35]. This task assesses empathy. Participants are shown videos and are required to make continuous ratings about the thoughts and feelings of the person in the video (called the target). Participant ratings are then compared to the target’s reported thoughts and feelings to index their accuracy. Mean ratings were used.
3. Mayer–Salovey–Caruso Emotional Intelligence Test (MSCEIT) [36]. This task assesses emotion management. Specifically, we used Branch 4, which includes two subtests—Social Management and Emotion Management. These tasks assess how participants manage others’ emotions and regulate their own emotions. Standard scores were used in the further analysis.

#### Interpersonal functioning

We used the Social Functioning Scale (SFS) [37] to measure interpersonal functioning. The SFS is a self-report scale designed to measure social skills and performance in social roles and includes seven subscales. For this study, we used subscales that measure behavior and engagement related to social situations; including social withdrawal/engagement (e.g., time spent alone, initiation of conversations, social avoidance), interpersonal behavior (e.g., number of friends, partner status, quality of communication), and pro-social activities (e.g., engagement in a range of common social activities).

### Statistical analysis

#### Exploratory graph analysis

We used a bootstrapped version of exploratory graph analysis (EGA) to explore links and communities of variables corresponding to social cognition, social motivation, general motivation, and interpersonal functioning. EGA was applied in *R* using the *EGAnet* package [27, 38]. EGA calculates Spearman correlations and uses the EBICglasso from the *qgraph* package [39] to estimate the sparse inverse covariance matrix. Spearman correlation was selected due to the ordinal character of social motivation variables and violation of normality assumptions. The EBICglasso function runs 100 values of the regularization parameter, generating 100 graphs, and the graph with the smallest Extended Bayesian Information Criteria (EBIC) is selected. Positive associations are displayed in green, and negative associations are displayed in red.

#### Network centrality

To quantify the relative importance of nodes within the network, we analyzed e*xpected influence* [40] and s*trength* [41] centrality metrics. Expected influence measures the relation of an item to others in the network and is the sum of partial correlations. Strength has similar properties to expected influence, but strength reflects the absolute sum of partial correlations. Thus, the main difference between these centrality metrics is that expected influence accounts for negative partial correlations and strength does not. Because negative correlations can lead to different centrality estimates, we reported both expected influence and strength. We used a Bootstrap approach on 5000 samples to evaluate the stability of these estimates. Other common centrality metrics, such as betweenness and closeness, were not reported due to concerns with stability [42]. Overall network stability was estimated using the Correlation stability coefficient (CS). We used recommended cutoffs for CS coefficients (CS > 0.25).

#### Community detection and community stability

EGAnet was again used to estimate communities. We used the Walktrap algorithm [43] was applied to find the number of dense subgraphs (i.e., communities) of the partial correlation matrix. The Walktrap algorithm measures similarities between vertices based on random walks, capturing the community structure. We then employed 1000 bootstrap samples, and EGA was applied in each.

To augment community detection results, we investigated item stability, defined as the proportion of times specific items clustered with their community across the replicated bootstrapped samples [38]. Low item stability will result in low structural consistency since the same items are unlikely to appear in the same community. In contrast, high item stability suggests that the community structure would replicate across different samples.

## Results

### Sample characteristics

The sample consisted of 147 patients with schizophrenia. Demographic and clinical characteristics are shown in Table 1.

**Table 1:** Descriptive statistics for demographic, clinical, and behavioral variables

	M (SD)
Demographics	
Age	42.86 (12.78)
% of males	69.39%
Years of education	13.24 (2.01)
Premorbid IQ	97.76 (11.25)
Psychopathology	
PANSS—Positive	14.99 (5.07)
PANSS—Negative	16.61 (6.12)
PANSS—General	30.14 (7.81)
General and social motivation	
BAS—Behavioral activation system*	13.51 (2.08)
BIS—Behavioral inhibition system	20.86 (4.23)
PANSS N4—Passive social withdrawal	2.95 (1.52)
PANSS G16—Active social avoidance	2.41 (1.46)
Interpersonal functioning	
SFS—Withdrawal	101.24 (9.86)
SFS—Communication	116.64 (17.14)
SFS—Prosocial behavior	111.77 (13.96)
Social cognition	
Empathic accuracy	0.49 (0.23)
MSCEIT—managing emotions	90.00 (10.95)
Penn emotion recognition task	29.94 (5.23)

*PANSS* Positive and Negative Schizophrenia Syndrome Scale, *SFS* Social Functioning Scale—presented as standard scores, *MSCEIT* Mayer–Salovey–Caruso Emotion Intelligence Test

^*^BAS was calculated as a mean score of three subscales

### Links between social cognition, social motivation, and interpersonal functioning

**Fig. 1:**
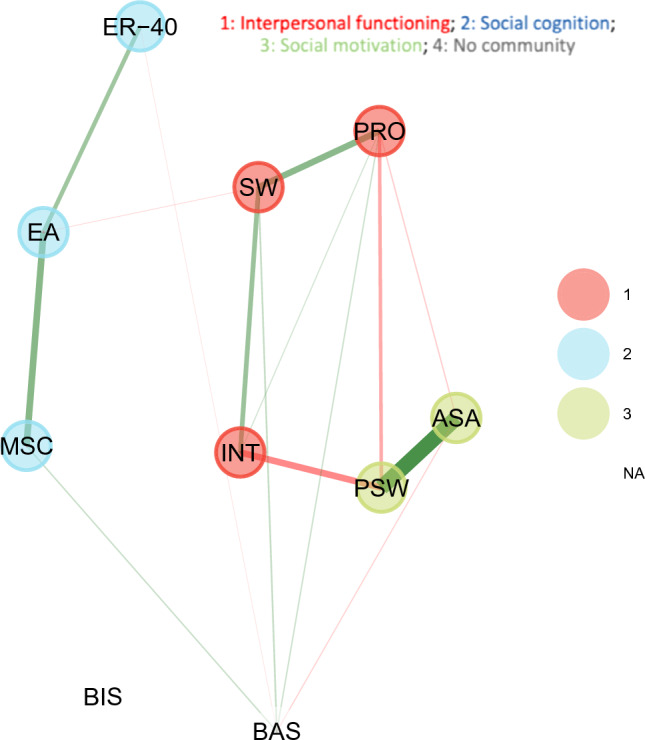
**Exploratory graph analysis with communities**. *PSW* passive social withdrawal, *ASA* active social avoidance, *PRO* prosocial activities, *SW* social withdrawal/engagement, *INT* interpersonal communication, *MSC* Mayer–Salovey–Caruso emotion intelligence test, *ER–40* penn emotion recognition test, *EA* empathic accuracy, *BIS* behavioral inhibition system, *BAS* behavioral activation system

The EGA model is shown in Fig. 1. Communities—nodes with the same color—are estimated based on the data and were not a priori determined. Regarding the links between social motivation and interpersonal functioning, active social avoidance showed a weak relationship with reduced prosocial activities. In addition to reduced prosocial activities, passive social withdrawal was also associated with lower interpersonal communication. Neither active social avoidance nor passive social withdrawal was associated with social withdrawal/engagement. The social cognition nodes had only negligible links to the interpersonal functioning nodes. Specifically, the only link was between empathic accuracy and social withdrawal/engagement. Additionally, there were no links between social cognitive and social motivation nodes.

### Links between general motivation, social motivation, and interpersonal functioning

BIS was not associated with any node in the network. In contrast, BAS was marginally linked to active social avoidance but was not linked to passive social withdrawal. Regarding interpersonal functioning, BAS was associated with social withdrawal/engagement and prosocial activities.

### Centrality of specific nodes

**Fig. 2:**
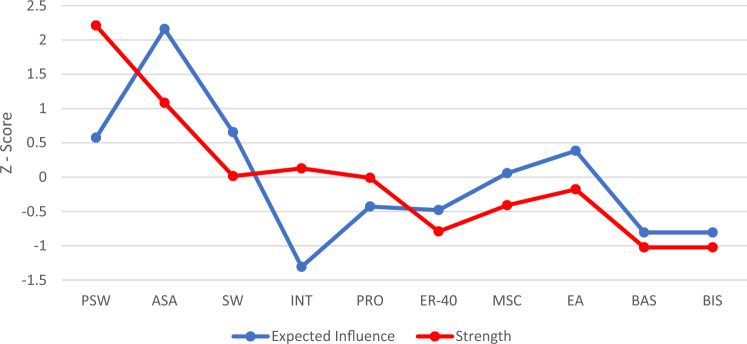
**Centrality measures**. *PSW* passive social withdrawal, *ASA* active social avoidance, *PRO* prosocial activities, *SW* social withdrawal/engagement, *INT* interpersonal communication, *MSC* Mayer–Salovey–Caruso emotion intelligence test, *ER–40* penn emotion recognition test, *EA* empathic accuracy, *BIS* behavioral inhibition system, *BAS* behavioral activation system

Centrality estimates were derived based on the magnitude of the relationship between nodes (Fig. 2). The nodes with the highest strength and expected influence across the network were active social avoidance and passive social withdrawal; however, this high centrality is partially due to their strong association, likely reflecting method variance. Despite BAS being connected to social cognitive, social motivation, and interpersonal functioning nodes, it (along with BIS) had the lowest strength and expected influence. CS coefficients evaluating network stability were sufficiently high (strength CS = 0.442; expected influence CS = 0.517) and were above recommended cut-off.

### Community detection and community stability

EGA estimated three distinct communities within the network. One community was formed from all social cognitive tasks. The strongest within-community association was between empathic accuracy (EA) and emotion management (MSC). A second community consisted of active social avoidance and passive social withdrawal. A third community emerged from all interpersonal functioning variables: social withdrawal/engagement, interpersonal communication, and prosocial activities. All nodes in that community were interconnected with all other nodes. Notably, the measures of general motivation, BIS and BAS, were not part of any community. The median number of estimated communities in bootstrap samples was 3 with 95% CI (1.96–4.04). Three communities emerged in 71% of samples. We evaluated the proportion of times that a particular community was replicated in bootstrap samples (structural consistency). Social motivation was replicated in 86% of bootstrapped networks, followed by social cognition (75%) and interpersonal functioning (61%).

**Fig. 3:**
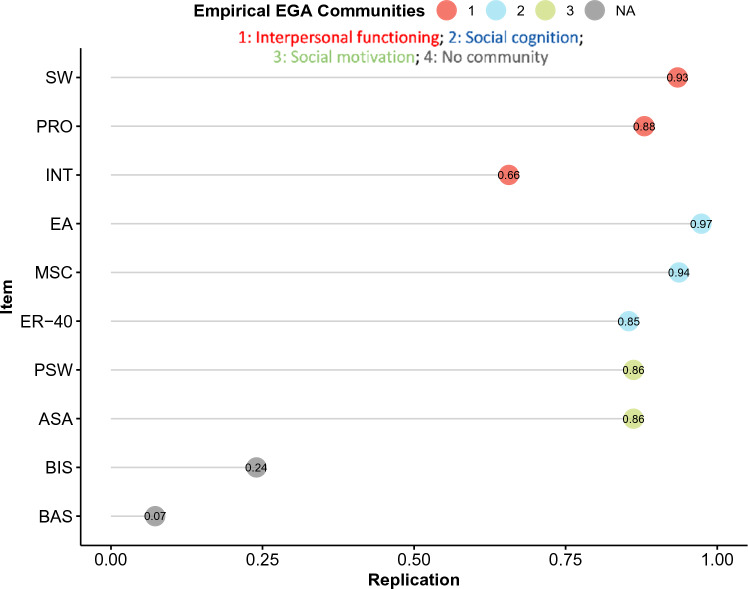
**Stability of estimated communities**. *PSW* passive social withdrawal, *ASA* active social avoidance, *PRO* prosocial activities, *SW* social withdrawal/engagement, *INT* interpersonal communication, *MSC* Mayer–Salovey–Caruso emotion intelligence test, *ER–40* penn emotion recognition test, *EA* empathic accuracy, *BIS* behavioral inhibition system, *BAS* behavioral activation system

The structural stability of communities was evaluated using 1000 bootstrap samples (see Fig. 3). On average, the social cognitive community (92%) and the social motivation community (86%) replicated well across the bootstrap samples. The Interpersonal functioning community also replicated to a sufficient level, with Interpersonal communication showing the lowest replication (66%) across samples. BIS and BAS were consistently unrelated to any of the three communities.

## Discussion

The goal of this study was to examine the unique associations among social motivation, general motivation, social cognition, and interpersonal functioning in people with schizophrenia. Our analyses yielded several intriguing findings. Social motivation was more interconnected with interpersonal functioning than social cognition, which showed minimal connections. Similarly, general motivation also showed minimal connections with interpersonal functioning and formed a separate community from social motivation. Together, these findings suggest that social motivation, specifically social approach motivation, is a key contributing factor to interpersonal functioning in schizophrenia.

Social approach and avoidance measured by PANSS were the most central nodes within the network. BIS showed the overall lowest importance with absence of strong links to other nodes. Centrality estimates analysis showed the importance of social motivation over general motivation and other nodes in the network. The importance of social withdrawal has been supported by recent network analytic studies linking motivation or negative symptoms to social functioning. The avolition–apathy factor was strongly linked to social functioning in individuals with schizophrenia and accounted for most of the variance [44], with similar results in patients with the first episode of psychosis [45]. Farina et al. [46] found that low social approach motivation has stronger impact on the functioning than heightened social avoidance. In addition, Robertson et al. [47], showed that passive social withdrawal explained substantially higher variability in general social functioning than active social avoidance in a large schizophrenia sample. Furthermore, these two motivational domains were better predictors of functional outcomes than social skills abilities. The pattern of results from the Robertson et al. study was later replicated in another study on an independent sample of individuals with schizophrenia [48]. Taking together, both social motivation factors are important, but research studies highlighted the central role of social approach motivation in schizophrenia for understanding daily problems in interpersonal relationships.

In the current study, social cognition showed only negligible links to interpersonal functioning when social motivation was controlled for. Social cognition nodes also showed lower centrality values. Indeed, the only association among these constructs in the EGA was between empathic accuracy and social withdrawal. Weaker links between social cognitive tasks and self-report of interpersonal functioning can be attributed to difficulties in precise estimating their own functioning (low introspective accuracy) [49]. Other well established social–cognitive measures might lead to different results [8]. Social motivation outperformed social cognition in explaining difficulties in interpersonal relationships. These results suggest the importance of interventions that focus on low approach motivation for interpersonal functioning in schizophrenia. For example, there is also evidence that Cognitive Enhancement Therapy leads to changes in negative symptoms, with the most significant improvement in passive social withdrawal [50].

Based on the community detection analyses, we found that trait-based behavioral inhibition was unrelated to state-based social motivation, social cognition, and interpersonal functioning. The absence of a link between active social avoidance and BIS was surprising given their conceptual similarities. On the contrary, the behavioral approach showed weak associations with social motivation, cognition, and interpersonal functioning. Taking together, our results support separation of general and social motivation in individuals with schizophrenia. The social motivation community was also reliably separated from social cognition, and interpersonal functioning. The separation from interpersonal functioning is particularly important as it supports a theoretical separation of core negative symptoms from social functioning.

The study had several limitations. The sample size (*n* = 147) is relatively small for network analyses. In addition, a range restriction in the PANSS on some items might limit generalization of results to more severely ill patients with prominent delusions and hallucinations. Despite that, we conducted bootstrap analyses and EGA item replication analysis to provide stability to our findings. Another limitation concerns our measures. To some extent, estimated communities and their interconnections might reflect share method variance problems (performance-based vs self-report vs clinician rated measures). Similarly, combining state (PANSS/SFS) and trait (BIS/BAS) measures could have influenced the strength of some relationships within and between estimated communities. The designation of PANSS as a state-based motivation measure also deserves mention. PANSS ratings are based on all available information during the last week, providing a limited estimate of state levels of social motivation. Future work could utilize a true state measure using in-the-moment data collection methods such as ecological momentary assessment [51]. Lastly, the study was limited in using single items measures of social motivation, which may have decreased variability and led to biased estimates [52].

In conclusion, we determined that motivational deficits, especially low social approach motivation, were more important than active social avoidance, trait general motivation, or even social cognition in understanding interpersonal functioning in schizophrenia. Based on the results of EGA, social motivation formed a community distinct from general motivation, social cognitive abilities, and interpersonal functioning. A major clinical implication of the current findings is that clinicians should routinely monitor state level of social motivation, both approach and avoidance, because of its relevance in understanding day-to-day difficulties of their patients in the interpersonal domain of social functioning. Based on these results, interventions for poor interpersonal functioning in schizophrenia can be very targeted (to enhancing social approach motivation, e.g., through motivational interviewing techniques that specifically target social engagement). The findings also point to future avenues of research that can investigate the mechanisms underlying poor social approach motivation in schizophrenia (e.g., with experimental paradigms, neuroimaging, etc.).
